# Magnetic Graphene Oxide Nanocarrier for Targeted Delivery of Cisplatin: A Perspective for Glioblastoma Treatment

**DOI:** 10.3390/ph12020076

**Published:** 2019-05-18

**Authors:** Sami A. Makharza, Giuseppe Cirillo, Orazio Vittorio, Emanuele Valli, Florida Voli, Annafranca Farfalla, Manuela Curcio, Francesca Iemma, Fiore Pasquale Nicoletta, Ahmed A. El-Gendy, Gerardo F. Goya, Silke Hampel

**Affiliations:** 1 Leibniz Institute of Solid State and Material Research Dresden, 01069 Dresden, Germany; samim@hebron.edu (S.A.M.); s.hampel@ifw-dresden.de (S.H.); 2 College of Pharmacy and Medical Sciences, Hebron University, Hebron 00970, Palestine; 3 Department of Pharmacy, Health and Nutritional Sciences, University of Calabria, Rende (CS), 87036 Rende, Italy; annafranca.farfalla@gmail.com (A.F.); manuela.curcio@unical.it (M.C.); francesca.iemma@unical.it (F.I.); fiore.nicoletta@unical.it (F.P.N.); 4 Children’s Cancer Institute, Lowy Cancer Research Centre, UNSW Sydney, Sydney 2031, Australia; OVittorio@ccia.org.au (O.V.); EValli@ccia.org.au (E.V.); FVoli@ccia.org.au (F.V.); 5 ARC Centre of Excellence for Convergent BioNano Science and Technology, Australian Centre for NanoMedicine, UNSW Sydney, Sydney 2052, Australia; 6 School of Women’s and Children’s Health, Faculty of Medicine, UNSW Sydney, Sydney 2052, Australia; 7 Department of Physics, University of Texas at El Paso, El Paso, TX 79968, USA; aelgendy@utep.edu; 8 Institute of Nanoscience of Aragon (INA) & Department of Condensed Matter Physics, University of Zaragoza, 50018 Zaragoza, Spain; goya@unizar.es

**Keywords:** magnetic targeting, graphene oxide, maghemite, glioblastoma, cisplatin

## Abstract

Selective vectorization of Cisplatin (CisPt) to Glioblastoma U87 cells was exploited by the fabrication of a hybrid nanocarrier composed of magnetic γ-Fe_2_O_3_ nanoparticles and nanographene oxide (NGO). The magnetic component, obtained by annealing magnetite Fe_3_O_4_ and characterized by XRD measurements, was combined with NGO sheets prepared via a modified Hummer’s method. The morphological and thermogravimetric analysis proved the effective binding of γ-Fe_2_O_3_ nanoparticles onto NGO layers. The magnetization measured under magnetic fields up to 7 Tesla at room temperature revealed superparamagnetic-like behavior with a maximum value of M_S_ = 15 emu/g and coercivity H_C_ ≈ 0 Oe within experimental error. The nanohybrid was found to possess high affinity towards CisPt, and a rather slow fractional release profile of 80% after 250 h. Negligible toxicity was observed for empty nanoparticles, while the retainment of CisPt anticancer activity upon loading into the carrier was observed, together with the possibility to spatially control the drug delivery at a target site.

## 1. Introduction

Malignant glioma is one of the most aggressive brain tumors, and the major cause of death from central nervous system cancers (median survival times less than 15 months from diagnosis) [1,2,3,4,5]. Glioma treatment is still one of the most difficult challenges for oncologists [6], and current therapies involve surgical intervention to achieve tumor debulking followed by adjuvant radio- and chemo-therapy [7]. Chemotherapy approaches are of paramount importance in the case of the most devastating and lethal grade IV glioma (Glioblastoma Multiforme, GBM), because the extensive tumor infiltration into the surrounding brain parenchyma makes surgery un-effective [8]. However, the therapeutic efficiency of chemotherapy is remains unsatisfactory for two main reasons: (i) the rare brain penetration of the anticancer agents systemically administered through the blood brain barrier (BBB) [9], and (ii) the poor glioma targeting of employed chemotherapeutics [10]. The latter issue is the main obstacle in the clinical treatment of Glioma with *cis*-diamminedichloroplatinum(II) (CisPt) [11], one of the most effective anticancer agents. CisPt suffers from a nonselective distribution between normal and tumor tissues, with the insurgence of severe adverse side effects, including acute nephrotoxicity, myelosuppression, and chronic neurotoxicity in adults [12,13,14], and lifelong health issues when the therapy was given in children [15,16]. Therefore, it is patently clear that, for an effective Glioma treatment, there is an urgent need for powerful and targeted CisPt delivery systems in order to promote preferential accumulation in cancer cells and thereby reduce the side effects [17]. Taking advantage of the peculiar features of tumor tissues such as the leaky neovasculature and the lack of functional lymphatic drainage, a wide range of nanoparticle drug carriers have been explored for this purpose [18].

Among others, graphene nanomaterials, mainly in the form of nanographene oxide (NGO), possess superior physicochemical, thermal, optical, mechanical, and biological properties [19,20,21]. NGO is widely explored for drug delivery applications by virtue of the large surface area (four times higher than that of any other nanomaterials) and the high stability of its water dispersion due to the richness of oxygen containing functional groups (e.g., carboxyl, epoxide, and hydroxyl groups) [22,23,24]. The suitability of NGO for the preparation of CisPt delivery vehicles with high loading efficiency is related to the presence of either the sp^2^-aromatic structure or the abundant oxidized sp^3^-portion on the edge, top, and bottom surfaces of each sheet [25,26,27], allowing the drug interaction through diverse mechanisms, including π-π stacking and hydrogen bonding [28,29,30,31,32,33,34,35].

More interestingly, functionalized NGO was found to highly accumulate in U87 human glioblastoma subcutaneous tumor xenografts [36,37], confirming that such nanocarriers can be considered a valuable tool for delivering CisPt to brain cancers. The efficiency of NGO delivery vehicles can be maximized by the incorporation of magnetic materials allowing the nanocarrier to be selectively driven into tumor tissues by the application of an external magnetic field [38]. In particular, magnetic nanoparticles based on iron oxide (maghemite γ-Fe_2_O_3_ or magnetite Fe_3_O_4_) were widely used for this purpose due to their biocompatibility and superparamagnetic properties [39,40]. The resulting NGO hybrid nanodevices were proposed as effective tools for glioblastoma treatment using Doxorubicin [41] and Irinotecan [42] as cytotoxic agents. Although possessing favorable properties for magnetic drug vectorization, the different chemical stabilities of Fe_3_O_4_ and γ-Fe_2_O_3_ may affect the toxicity of the delivery vehicle [43]. The lower chemical stability of Fe_3_O_4_ resulted in the release of Fe^2+^ ions from the nanoparticle cores, which can catalyze the formation of reactive oxygen species (ROS) damaging cell membrane and organelles, with the insurgence of adverse long-term side effects [44]. On the other hand, γ-Fe_2_O_3_ was found to be a better material owing to either the magnetic features or the high chemical stability [45].

In the present study we explored the possibility to employ NGO–Iron oxide nanohybrids (γ-Fe_2_O_3_@NGO) as a CisPt carrier for glioblastoma treatment by intercalating γ-Fe_2_O_3_ nanoparticles into NGO sheets. After characterizing the physical, chemical, and morphological properties, CisPt was loaded onto the nanocarrier for several drug-to-carrier ratios and their cytotoxicity was tested on human U87 cell lines.

## 2. Results and Discussion

### 2.1. Properties of γ-Fe_2_O_3_@NGO Nanohybrid

As previously reported, the size of NGO is a parameter that strongly affects the drug delivery effectiveness of NGO-based systems in vitro and in vivo [46,47]. Specifically, low-sized NGOs (lateral dimension ≈100 nm) have been reported to have the best performance [46].

**Figure 1:**
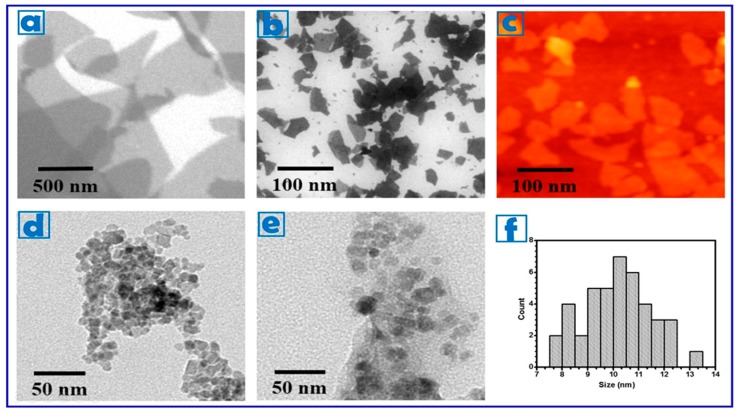
SEM images of (**a**) GO; and (**b**) NGO showing an average lateral width of 350–400 and 80–100 nm, respectively. (**c**) AFM image of NGO. TEM images of (**d**) γ-Fe_2_O_3_; and (**e**) γ-Fe_2_O_3_@NGO nanoparticles. (**f**) Size distribution of γ-Fe_2_O_3_ nanoparticles (approximately 10 nm).

The average size of our graphite oxide (GO) particles, as assessed by scanning electron microscopy (SEM), revealed an average size (lateral width) of 350–400 nm. These particles were therefore subsequently sonicated until NGO with lateral width of 80–100 nm and a thickness of 6.3 nm was attained (10 NGO sheets, assuming an interlayer distance of 0.7 nm) [48] (Figure 1a–c).

**Figure 2:**
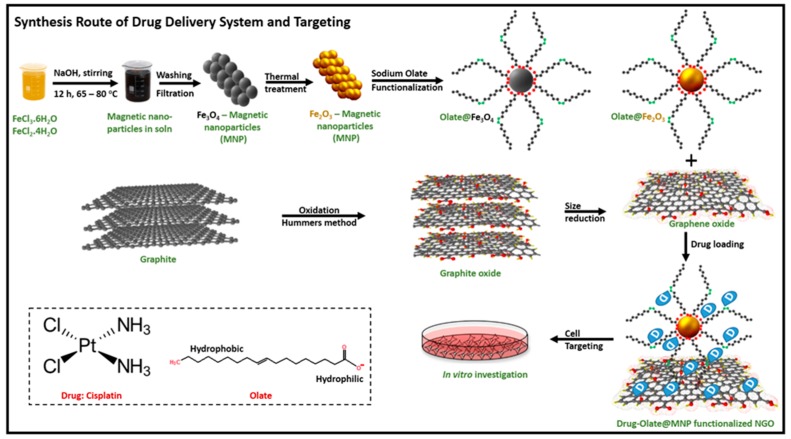
Schematic representation of the preparation of γ-Fe_2_O_3_@NGO.

The obtained NGO 100 nm were employed for the preparation of the magnetic hybrid device (γ-Fe_2_O_3_@NGO) as sketched in Figure 2.

Maghemite (γ-Fe_2_O_3_) nanoparticles were chosen to provide magnetic properties to the nanohybrids because of their high chemical stability, biocompatibility, and large magnetic moment at room temperature in its bulk form [40]. Superparamagnetism is crucial for application in biomedicine, because, despite the strong response to an external magnetic field, the absence of residual magnetic properties upon removal of the external field prevents nanoparticles from aggregation in biological environment [49,50,51,52]. γ-Fe_2_O_3_ nanoparticles (average size of 10 nm, see Figure 1d,f) were synthesized by annealing of magnetite Fe_3_O_4_ prepared by a chemical co-precipitation technique of FeCl_3_ and FeCl_2_ solutions [53,54], and then coated with oleic acid/sodium oleate to enhance their dispersion in water media and thus the biocompatibility features [55]. Since previously reported data proved the presence of transport systems importing fatty acids into the brain with high affinity and efficiency, it is reasonable to hypothesize that this coating strategy could be appropriate for targeting the blood brain barrier [56].

**Figure 3:**
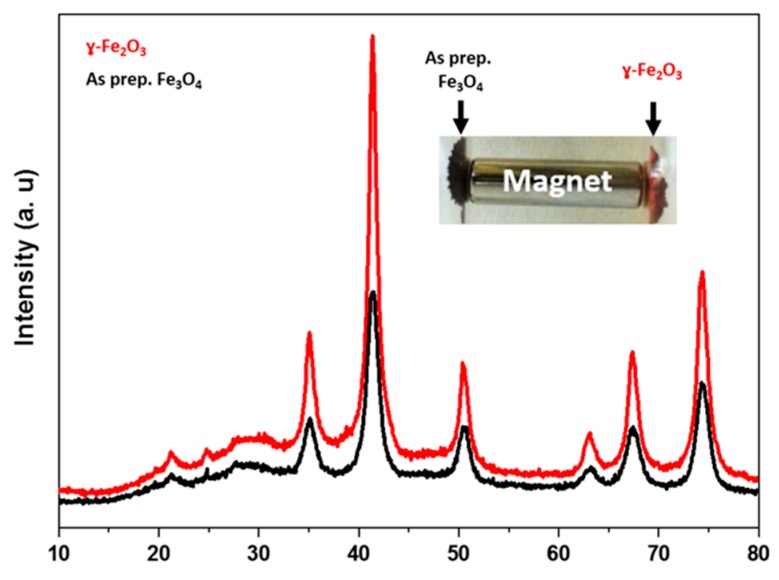
XRD patterns for Fe_3_O_4_ and γ-Fe_2_O_3_.

Despite the evidence of Fe_3_O_4_ to γ-Fe_2_O_3_ oxidation from the change of color of the sample (from black to reddish-brown color, see Figure 3), we investigated this phase change by XRD measurements. Figure 3 showed the XRD patterns of both compounds, and the *d*-spacing values emulated well the data deduced from the Joint Committee on Powder Diffraction Standards (JCPDS) cards 19-629 (Fe_3_O_4_) and 39-1346 (γ-Fe_2_O_3_).

The result indicated no major differences between the two patterns, in each set of XRD patterns, the crystalline structure of magnetite and/or maghemite with indexes (hkl) ascribed to (220), (311), (400), (422), (511), and (440) were observable at the diffraction angels 2θ = 35.1°, 41.4.6°, 50.4°, 63.1°, 67.4° and 74.3° crystal planes, respectively. This result indicated that the thermal treatment of as prepared Fe_3_O_4_ produced γ-Fe_2_O_3_ (maghemite) crystal form [57].

**Figure 4:**
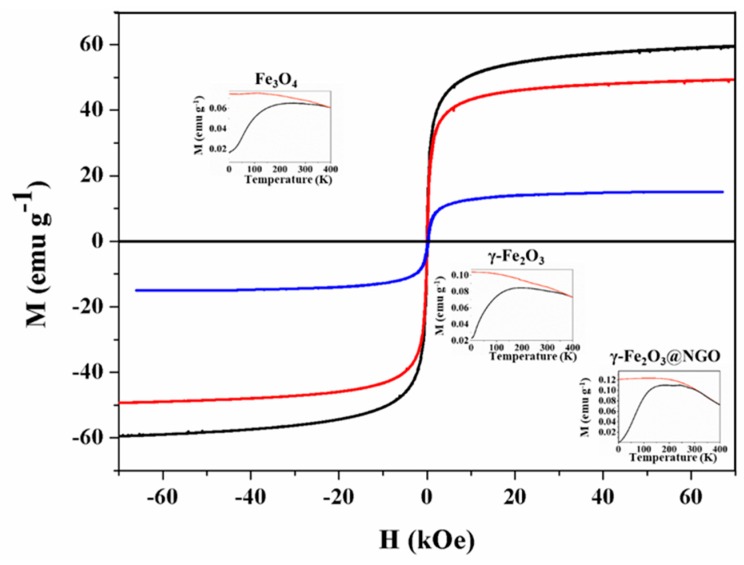
Hysteresis loops M(H) for Fe_3_O_4_ (black) and γ-Fe_2_O_3_ (red) and γ-Fe_2_O_3_@NGO (blue) nanoparticles. The insets show the Zero-field cooled (black) and field-cooled (orange) magnetization curves for Fe_3_O_4_, γ-Fe_2_O_3_, and γ-Fe_2_O_3_@NGO, taken with H_FC_ = 100 Oe.

The magnetization vs. field M(H) curves for the annealed γ-Fe_2_O_3_ nanoparticles showed nearly closed hysteresis loops, with zero coercivity (Figure 4).

The magnetization did not fully saturate within our experimentally available fields (H = 70 kOe), attaining a value of MS = 59.36 emu/g and MS = 49.25 emu/g at H = 70 kOe for Fe_3_O_4_ and γ-Fe_2_O_3_, respectively. After assembling γ -Fe_2_O_3_ into NGO the Ms value was 15.02 emu/g, consistent with a ≈30.5% wt. of magnetic material into NGO matrix, confirming the dispersion on magnetic nanoparticle into the hybrid platform. The coercivity values at room temperature were HC ≈ 0 for all samples. The zero field cooling (ZFC) and field cooling (FC) curves at H_FC_ = 100 Oe of Fe_3_O_4,_ γ-Fe_2_O_3_ and γ-Fe_2_O_3_@NGO samples reflected similar features, i.e., a broad maximum in the ZFC curves originated from the distribution of blocking temperatures due to the distribution of particle sizes (see inset of Figure 4). The maxima were centered around T ≈ 194, 245, and 242 for Fe_3_O_4_, γ-Fe_2_O_3_, and γ-Fe_2_O_3_@NGO, respectively. These broad maxima are consistent with the blocking of the smallest nanoparticles at these temperatures, while the presence of irreversible behavior up to the highest temperature (400 K) suggests that a fraction of the largest particles are still blocked above room temperature. 

**Figure 5:**
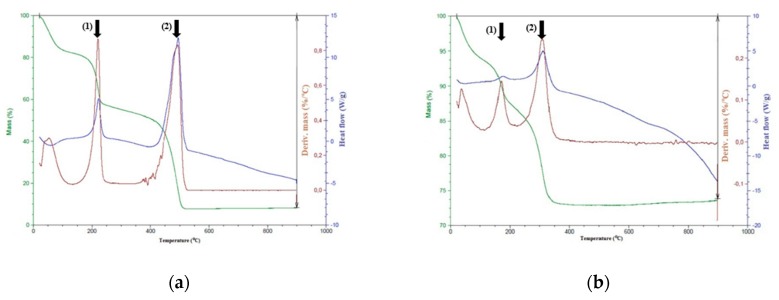
TGA curves for NGO (**a**) and γ-Fe_2_O_3_@NGO (**b**).

The thermogravimetric analysis (TGA) curves of NGO and γ-Fe_2_O_3_@NGO were depicted in Figure 5.

For the NGO sample (Figure 5a), the mass loss in the range 150–250 °C with the maximum in the derivative %M/°C graph at 215 °C (arrow (1)) was ascribed to the decomposition of decorated oxygen functionalities on the basal graphene structure, while between 400 and 525 °C (maximum at 490, see arrow (2)), a high weight loss occurs due to the discard of more thermally stable oxygen groups. On the other hand, for γ-Fe_2_O_3_@NGO (Figure 5b), these mass losses were found to shift to lower temperatures (maximum in the derivative %M/°C graph at 170 °C and 305 °C, respectively) as a consequence of the effective binding of γ-Fe_2_O_3_ nanoparticles onto NGO layers.

### 2.2. Evaluation of Carrier Performances

**Figure 6:**
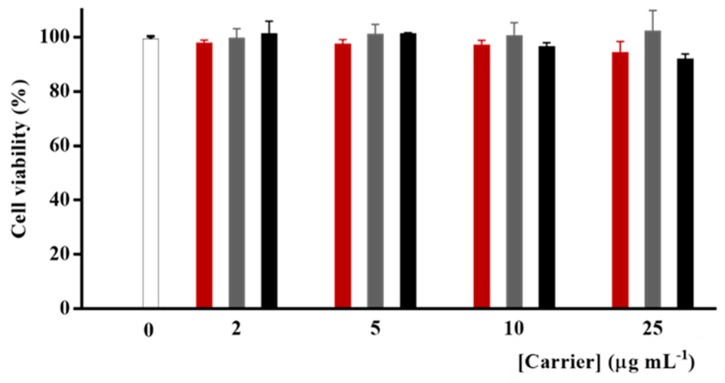
U87 viability after treatment with empty γ-Fe_2_O_3_ (**red**) and NGO (**grey**) and γ-Fe_2_O_3_@NGO (**black**).

Before testing the efficiency of γ-Fe_2_O_3_@NGO nanohybrid as CisPt carrier, we evaluated the toxicity of the empty nanoparticles (γ-Fe_2_O_3_, NGO, and γ-Fe_2_O_3_@NGO) on human glioblastoma U87 cell lines at a concentration range of 0–25 µg mL^−1^. This range of concentration was selected because of the absence of any sign of aggregation as per Dynamic light-scattering (DLS) measurements. The viability values (>96% for all samples and concentrations, see Figure 6) proved the high biocompatibility of all nanoparticle systems, confirming their suitability as drug carrier [58].

The ultimate aim of the study is to check the suitability of γ-Fe_2_O_3_@NGO to selectively vectorize the cytotoxic drug to the tumor site under magnetic actuation. Indeed a key requirement for this nanocarrier is the ability to retain the drug until it reaches the target site. γ-Fe_2_O_3_@NGO was found to possess high affinity for CisPt (Drug Loading Efficiency of 0.37 mg mg^−1^) and the release profiles were recorded after loading the drug by a soaking procedure (drug to carrier ratio of 10% by weight).

**Figure 7:**
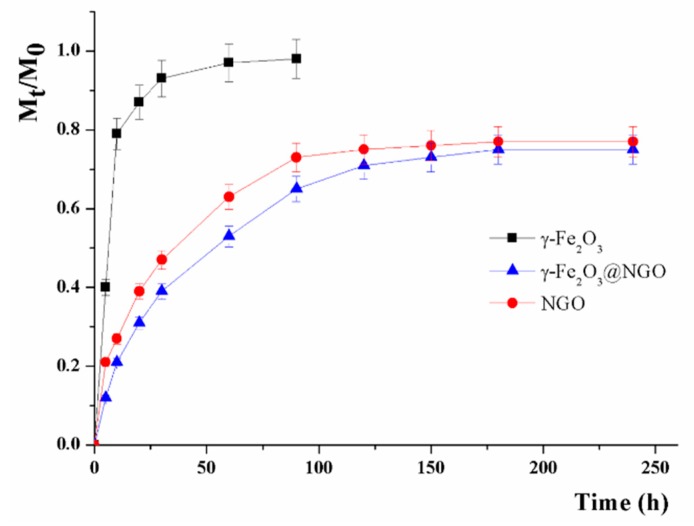
CisPt release profiles from γ-Fe_2_O_3_@NGO, γ-Fe_2_O_3_, and NGO.

The cumulative amount of drug released (M_t_/M_0_) was compared with those recorded when uncombined γ-Fe_2_O_3_ or NGO were employed as carrier (Figure 7).

For a more exhaustive analysis of the CisPt release profiles, a mathematical model considering the partition between the carrier and the surrounding environments and the underlying mechanism of the drug release was applied according to the literature [59]. In this model, a key parameter (α) was adopted to describe the physicochemical affinity of the drug between the carrier and solvent phases according to Equation (1):

(1) $$\alpha =\frac{F_{max}}{1-F_{max}}$$

where *F_max_* represents the maximum value of relative release (*M_t_/M*_0_).

The overall drug release can be modeled according to reversible first- or second-order kinetics of Equations (2) and (3).

(2) $$\frac{M_{t}}{M_{0}}=F_{max}\left(1-e^{-\left(\frac{k_{R}}{F_{max}}\right)t}\right)$$

(3) $$\frac{M_{t}}{M_{0}}=\frac{F_{max}\left(e^{2\left(\frac{k_{R}}{\alpha }\right)t}-1\right)}{1-2F_{max}+e^{2\left(\frac{k_{R}}{\alpha }\right)t}}$$

with *k_R_* being the release rate constant.

The time required for reaching 50% of *F_max_* ($t_{1/2}$) can be obtained by applying the following Equations (4) and (5), respectively:

(4) $$t_{1/2}^{1}=\frac{F_{max}}{k_{R}}ln2$$

(5) $$t_{1/2}^{2}=\frac{\alpha }{2kR}\ln\left(3-2F_{max}\right)$$

Both models are suitable for describing the CisPt release (see R^2^ in Table 1), with the presence of NGO making the release better described by reversible second-order kinetics. In the absence of NGO, a fast CisPt release was recorded (M_t_/M_0_ of 0.90 after 20 h), with high α value indicating a low affinity of the drug towards the carrier phase (γ-Fe_2_O_3_). On other hand, the strong interaction between CisPt and NGO [60,61,62] resulted in a more extended release over time (F_max_ < 0.8 even after 250 h), with the same affinity (3.54) recorded for either NGO or γ-Fe_2_O_3_@NGO. The presence of γ-Fe_2_O_3_ in γ-Fe_2_O_3_@NGO was found to slow the release, with reduced kinetic constant (k_R_) and t_1/2_ values moving from 19.01 (NGO) to 29.38 (γ-Fe_2_O_3_@NGO) h. This could be ascribed to the hindrance to the drug diffusion from the NGO to the solvent phase by the oleate coating of γ-Fe_2_O_3_ nanoparticles [55].

**Figure 8:**
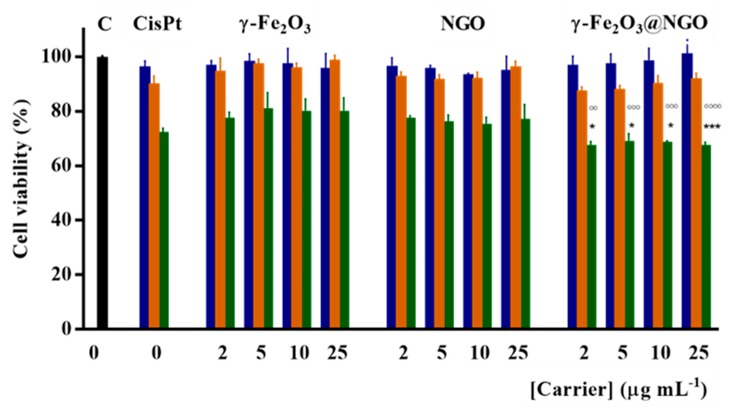
U87 viability after 72 h incubation with CisPt concentrations 2.5 (**blue**); 5.0 (**orange**); and 10.0 (**green**) µM in the free form and after loading on γ-Fe_2_O_3_; NGO; γ-Fe_2_O_3_@NGO. Carrier concentrations were 2.0; 5.0; 10.0; and 25.0 µg mL^−1^. An overall *p*-value less than 0.05 was accepted as significant. For individual comparisons of γ-Fe_2_O_3_@NGO (10 µM CisPt) vs. γ-Fe_2_O_3_ or NGO at the same concentrations, adjusted *p*-values are indicate as * *p* < 0.05 vs. NGO; *** *p *< 0.001 vs. NGO; °°° *p* < 0.001 vs. γ-Fe_2_O_3_; °°°° *p* < 0.0001 vs. γ-Fe_2_O_3_. Error bars represent standard error of the mean (n = 3 independent experiments).

CisPt loaded γ-Fe_2_O_3_@NGO were employed in different drug-to-carrier ratios (concentration ranges of 0–25 µg mL^−1^ and 0–10 µM for carrier and drug, respectively, see Figure 8). From the data in Figure 8, it is clear that the lowest toxic concentrations of CisPt (10 µM) is unchanged after loading on the different carriers, with γ-Fe_2_O_3_@NGO being the most effective vehicle for killing cells.

**Figure 9:**
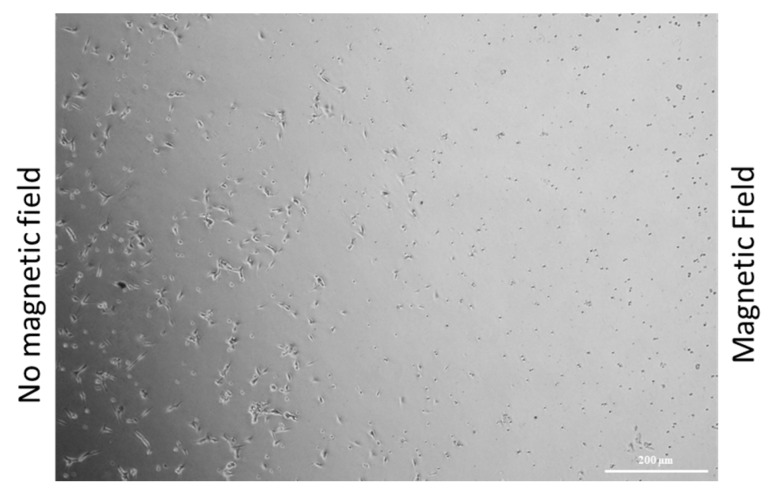
Optical microscope image U87 cells incubated with 10 µM CisPt loaded γ-Fe_2_O_3_@NGO under the effect of a permanent magnet.

To investigate the possibility of obtaining a selective vectorization of the drug, a proof of concept experiment was designed by incubating U87 cells with 10 µM CisPt loaded γ-Fe_2_O_3_@NGO for 24 h kept under the effect of a magnetic field generated by a permanent Nd-Fe-B magnet. As a result of the magnetic carrier driven spatial concentrations of the drug, a selective cell death at the region close to the magnet was reached, even at low drug concentration (10 µM), with no relevant toxicity detected on the region where the magnetic forces were negligible (Figure 9).

Overall, the obtained results are of great interest for application in cancer therapy for two main outcomes: (i) an effective magnetic vectorization of CisPt to cancer cells can be reached, since a very low amount of CisPt was released in the first 20 h (M_t_/M_0_ < 0.30) and thus negligible side toxicity can be hypothesized, and (ii) the CisPt loaded into γ-Fe_2_O_3_@NGO is biologically active in reducing the viability of cancerous with an efficiency comparable with that of the free drug.

Future experiments will be performed for evaluating the therapeutic performance of the designed magnetic nanohybrid, by determining the pharmacokinetics profiles with or without a magnetic field, the anticancer activity in appropriate in vivo models, and the possibility to use the system for theranostics applications.

## 3. Materials and Methods 

### 3.1. Synthesis of Graphite Oxide

Graphite oxide particles were prepared from graphite powder (natural, -200 mesh, 99.9995% purity, Alfa Aesar) by using a modified Hummers method [63]. Graphite powder (1.0 g) was sonicated in water for 5 min, filtrated, washed with water and dried in an oven at 40 °C for 12 h. The dried graphite was transferred to a beaker and mixed with concentrated H_2_SO_4_ (98%, 23 mL). The mixture was left overnight under stirring at room temperature. Thereafter, 3.0 g KMnO_4_, as an oxidizing agent, was added gradually while keeping the reaction mixture below 10 °C, in order to decorate the surfaces of graphite by various oxygen groups (hydroxy, epoxy, carboxylic, etc.). After complete addition of KMnO_4_, the reaction mixture was stirred for 30 min at 35 °C and 45 min at 50 °C for enhancing the degree of oxidation. 46.0 mL of distilled water was added while maintaining the temperature between 98–105 °C for 30 min 10 mL of 30% H_2_O_2_ was added in order to terminate the reaction. The mixture of GO was washed several times with 5% HCl and water during the suction filtration. The filtrated graphite oxide was dried in an oven at 40 °C for 5 h.

### 3.2. Synthesis of Nanographene Oxide

NGO particles were prepared as reported previously [46]. The resultant material of graphite oxide was cracked in distilled water with different power percent and sonication time using a horn-tipped ultrasonic probe. The material was separated to different sizes by repeated centrifuge and filtration. SEM images were obtained using a FEI, NOVA NanoSEM200 (FEI, Hillsboro, OR, USA) with an acceleration voltage of 15 kV. AFM images of well-defined NGO sizes were acquired using Digital Instruments Veeco, NanoScope IIIa, operating in the tapping mode. The images were analyzed using WSxM software designed by Nanotech Electronica (Madrid, Spain). The distribution used during this study was approximately 100 nm in lateral size and 6 nm in thickness.

### 3.3. Synthesis of Maghemite Nanoparticles

Maghemite γ-Fe_2_O_3_ nanoparticles were synthesized in a three-step procedure as follows: first, the magnetite Fe_3_O_4_ nanoparticles were prepared by co-precipitation method in basic medium [53]. The synthesis of Fe_3_O_4_ nanoparticles is shown in Equation (6):

2FeCl_3_ + FeCl_2_ + 8NaOH → Fe_3_O_4_ (s) + 4H_2_O + 8NaCl (6)

Briefly, 2.25 g of FeCl_3_·6H_2_O and 0.825 g of FeCl_2_·4H_2_O were mixed in an alkaline solution (NaOH, 1.7 g). The mixture stirred at 65–80 °C for 12 h. The resultant material was filtrated and washed many times by distilled water and ethanol.

Subsequently, magnetite Fe_3_O_4_ nanoparticles were employed as starting material for the synthesis of maghemite γ-Fe_2_O_3_. An initial amount of 1.0 g of Fe_3_O_4_ was placed in furnace and heated up to 450 °C in the presence of Argon and H_2(g)_ for 12 h [54,57]. Thereafter, the reaction was quenched down to room temperature. The resulting material was collected, washed several times in deionized water and ethanol, dried in an oven at 65 °C for 3 h. A second annealing was applied at the same conditions in order to identify the structure of Fe_2_O_3_ whether it was maghemite or hematite form.

In the final step, 0.5 g of γ-Fe_2_O_3_ were heated to 60 °C for 15 min separately. Consequently, an excess of sodium oleate (20% wt/vol) was added under vigorous stirring for 15 min. Oleate functionalized nanoparticles were collected by magnetic decantation to remove the non-magnetic materials. The product was washed with water and acetone several times, filtrated and dried in an oven at 40 °C for 2 h.

The relevant X-ray diffraction patterns were performed by using Pert Pro MPD PW3040/60 X-ray diffractometer with Co K_α_ radiation (λ = 0.179278 nm) at ambient temperature.

### 3.4. Synthesis of γ-Fe_2_O_3_@NGO Nanohybrid

An amount of 0.5 g of NGO -100 nm particles was sonicated for 15 min in order to homogenize it in distilled water. The solution was heated up to 60 °C for 15 min directly; an excess of γ-F_e2_O_3_ system was added and stirred for 15 min. The final material was separated by magnetic decantation, washed with water and acetone, filtrated and dried in an oven at 40 °C for 2 h. TEM images were recorded on HRTEM/Tecnai F30 [300 kV] (FEI, Hillsboro, OR, USA). TGA was performed on a STA 409 PC/PG-Luxx analyzer (Netzsch, Selb, Germany). Measurements were conducted in a nitrogen atmosphere (flow of 10 mL min^−1^), with an initial sample weight of ∼10 mg in the temperature range 50–900 °C at a heating rate of 10 °C min^−1^.

Drug loading efficiency (DLE) of γ-Fe_2_O_3_@NGO for CisPt was estimated by mixing drug and carrier in a 1:1 ratio (by weight) and determining the amount of unloaded CisPt by UV-Vis on a Jasco V-530 UV/Vis spectrometer (Jasco Europe s.r.l., Milan, Italy) at 301 nm [64]. DLE was calculated according to the following Equation (7):

(7) $$DLE\;\left(mg\;mg^{-1}\right)=\frac{W_{D}}{W_{C}}$$

where *W_D_* and *W_C_* are the amount of loaded drug and carrier, respectively. In our condition, to ensure the same amount of drug being loaded on the three carriers (γ-Fe_2_O_3_@NGO, γ-Fe_2_O_3_, or NGO) the CisPt loading procedure was performed by mixing, in separate experiments, variable amounts of CisPt solution with the carriers and drying the products under vacuum at RT.

### 3.5. Magnetic Characterization

Magnetization curves were measured as function of temperature M(T) in the 4 K ≤ T ≤ 400 K temperature rage, in a SQUID magnetometer (MPMS 5000 from Quantum Design). The Zero-field-cooling (ZFC) and Field-cooling (FC) curves were measured under a field-cooling field H_FC_ = 100 Oe Hysteresis loops M(H) were taken at 4 K and 300 K within the −70 kOe ≤ H ≤ +70 kOe field range. For all these measurements, the colloids were conditioned in cylindrical sample holders and diamagnetic signal were extracted from the total magnetization.

### 3.6. In Vitro Cisplatin Release

Release experiments were performed by dialysis methods using 5.0 mL phosphate buffer saline (10^−3^ M, pH 7.4) was releasing media and dialysis tubing cellulose membranes of 25 mm average flat width and 12,000 MW cutoff (Fisher Scientific, Waltham, MA, USA). 5.0 mg nanoparticles (γ-Fe_2_O_3_@NGO, γ-Fe_2_O_3_, and NGO) loaded with CisPt were inserted into the dialysis tubes and subject to dialysis. At predetermined time intervals, the amount of CisPt in the releasing media was determined by UV-Vis on a Jasco V-530 UV/Vis spectrometer (Jasco Europe s.r.l., Milan, Italy) at 301 nm [64]. The cumulative amount of drug released (*F*) was calculated using the following Equation (8):

(8) $$F=\frac{M_{t}}{M_{0}}$$

where *M_t_* and *M*_0_ are the amounts of drug in solution at time t and loaded into the carrier, respectively. Sink conditions were maintained through the experiment: the maximal theoretical concentration of dissolved CisPt was 0.33 mM, with its solubility being 3.3 mM in these conditions [65].

### 3.7. Cell Growth Inhibition Assays

Human Glioblastoma cells (U87) were grown as a monolayer in a humidified atmosphere at 37 °C and in 5% CO_2_ in the presence of Dulbecco’s Modified Eagle Medium (DMEM) supplemented with 10% Fetal bovine serum (FBS), 1% L-glutamine, and 1% penicillin–streptomycin. Treatment effects on U87 cell growth were measured on the basis of the metabolic activity of cells using Alamar Blue assays [66]. Briefly, cells were plated in clear transparent 96-well plates at an optimized cell density of 2.5 × 10^3^ cells per well 48 h prior to treatment. Cells were then treated with either CisPt loaded or unloaded carriers (γ-Fe_2_O_3_@NGO, γ-Fe_2_O_3_, NGO) and effects on cell growth assessed 72 h later. Treatments involved the combination of CisPt and carrier concentrations of 2.5; 5.0; 10.0 µM and 2.0; 5.0; 10.0; 25.0 µg mL^−1^, respectively. Resazurin reduction was measured (excitation 530 nm, emission 590 nm) on a Versamax microplate reader (Molecular Devices, Sunnyvale, CA, USA).

To evaluate the magnetic vectorization ability, viability experiments were performed by treating 250 X 10^3^ cells seeded in a 35 mm petri dish with 10 µM CisPt loaded on γ-Fe_2_O_3_@NGO for 24 h under the effect of a magnetic field generated by a permanent magnet (100 G).

All chemicals were purchased by from Merck/Sigma Aldrich, Taufkirchen, Germany.

### 3.8. Statistical Analysis

Three experiments were carried out in triplicate. Values were expressed as means ± standard error of the mean. For viability assay, statistical significance was assessed by one-way analysis of variance followed by post-hoc comparison test (Tukey’s test). Significance was set at *p* < 0.01.

## 4. Conclusions

The possibility of CisPt delivery to specific target sites by remote actuation was reached by combining γ-Fe_2_O_3_ magnetic nanoparticles ensembled into a NGO nanoplatform. The correct assembly of the components was responsible for the efficiency of γ-Fe_2_O_3_@NGO as a drug delivery system. While NGO conferred high loading capabilities to the nanosystems, the magnetic nanoparticles provided the magnetic actuation capabilities for targeting and delivery of therapeutics.

The mathematical model of the CisPt release profiles suggested a sustained reversible second-order kinetics, which implies low amounts of CisPt released during the first seconds of the experiments. This type of release profile is of major importance if low toxicity levels are required for in vivo applications.

These findings, considered together with the retainment of CisPt toxicity upon loading and the possibility to increase the dose delivered at the target site by a magnetic actuation, make the nanocarrier developed here a valuable tool for applications in cancer therapy.

## Figures and Tables

**Table 1:** R^2^ values and kinetic parameters for CisPt release according to the applied mathematical model.

Mathematical Model	Parameter	γ-Fe_2_O_3_	NGO	γ-Fe_2_O_3_@NGO
$\frac{M_{t}}{M_{0}}=\;F_{max}\left(1-e^{-(k_{R}/M_{max})t}\right)$	R^2^	0.9818	0.9822	0.9909
	Fmax	0.98	0.76	0.74
	α	49	3.17	2.85
	k_R_ (10^−2^)	12.71	2.76	1.85
	$t_{1/2}^{1}$ (h)	5.35	18.81	27.00
$\frac{M_{t}}{M_{0}}=\frac{F_{max}\left(e^{2\left(\frac{k_{R}}{\alpha }\right)t}-1\right)}{1-2F_{max}+\;e^{2\left(\frac{k_{R}}{\alpha }\right)t}}$	R^2^	0.9340	0.9908	0.9960
	Fmax	0.97	0.78	0.78
	α	32.33	3.54	3.54
	k_R_ (10^−2^)	18.28	3.42	2.25
	$t_{1/2}^{2}$ (h)	5.15	19.01	29.38
